# Hair cortisol levels, psychological stress and psychopathological symptoms as predictors of postpartum depression

**DOI:** 10.1371/journal.pone.0182817

**Published:** 2017-08-28

**Authors:** Rafael A. Caparros-Gonzalez, Borja Romero-Gonzalez, Helen Strivens-Vilchez, Raquel Gonzalez-Perez, Olga Martinez-Augustin, Maria Isabel Peralta-Ramirez

**Affiliations:** 1 Brain, Mind and Behavior Research Center (CIMCYC), Faculty of Psychology, University of Granada. Granada. Spain; 2 Gynecology and Obstetrics Department, Hospital de Poniente, El Ejido, Spain; 3 Midwifery Department, Gongora Primary Health Center, Granada, Spain; 4 Department of Pharmacology, CIBERehd, School of Pharmacy, Instituto de Investigación Biosanitaria ibs.GRANADA, University of Granada, Granada, Spain; 5 Department of Biochemistry and Molecular Biology II, CIBERehd, School of Pharmacy, Instituto de Investigación Biosanitaria ibs.GRANADA, University of Granada, Granada, Spain; Klinikum der Johann Wolfgang Goethe-Universitat Frankfurt, GERMANY

## Abstract

Postpartum depression affects a huge number of women and has detrimental consequences. Knowing the factors associated with postpartum depression during pregnancy can help its prevention. Although there is evidence surrounding behavioral or psychological predictors of postpartum depression, there is a lack of evidence of biological forecasters. The aim of this study was to analyze the sociodemographic, obstetric, and psychological variables along with hair cortisol levels during the first, second, and third trimesters of pregnancy that could predict postpartum depression symptoms. A sample of 44 pregnant women was assessed during 3 trimesters of pregnancy and the postpartum period using psychological questionnaires and hair cortisol levels. Participants were divided into 2 groups: a group with postpartum depression symptoms and a group with no postpartum depression symptoms. Results showed significant positive differences between groups in the first trimester regarding the Somatization subscale of the SCL-90-R (*p* < .05). In the second trimester, significant differences were found in the Somatization, Depression, Anxiety, and GSI subscales (*p* < .05). In the third trimester significant differences between both groups were found regarding pregnancy-specific stress. We found significant positive differences between groups regarding hair cortisol levels in the first and the third trimester. Hair cortisol levels could predict 21.7% of the variance of postpartum depression symptoms. In conclusion, our study provided evidence that psychopathological symptoms, pregnancy-specific stress, and hair cortisol levels can predict postpartum depression symptoms at different time-points during pregnancy. These findings can be applied in future studies and improve maternal care in clinical settings.

## Introduction

Although mental health problems during the postpartum period often go unrecognized and untreated, the National Institute for Health and Care Excellence [1] recommends an urgent intervention due to potential detrimental effects on the newborn and the mother’s life. Postpartum depression affects from 10% to 15% of women after delivery and consists of emotional liability and sometimes suicidal ideation [2]. An extensive number of studies have shown an association between postpartum depression and poor bonding between the mother and the newborn as well as lower infant neurodevelopment [3, 4]. Early detection of factors associated with postpartum depression can prevent its appearance and negative outcomes (e.g. postpartum psychosis) [5].

**Fig 1:**
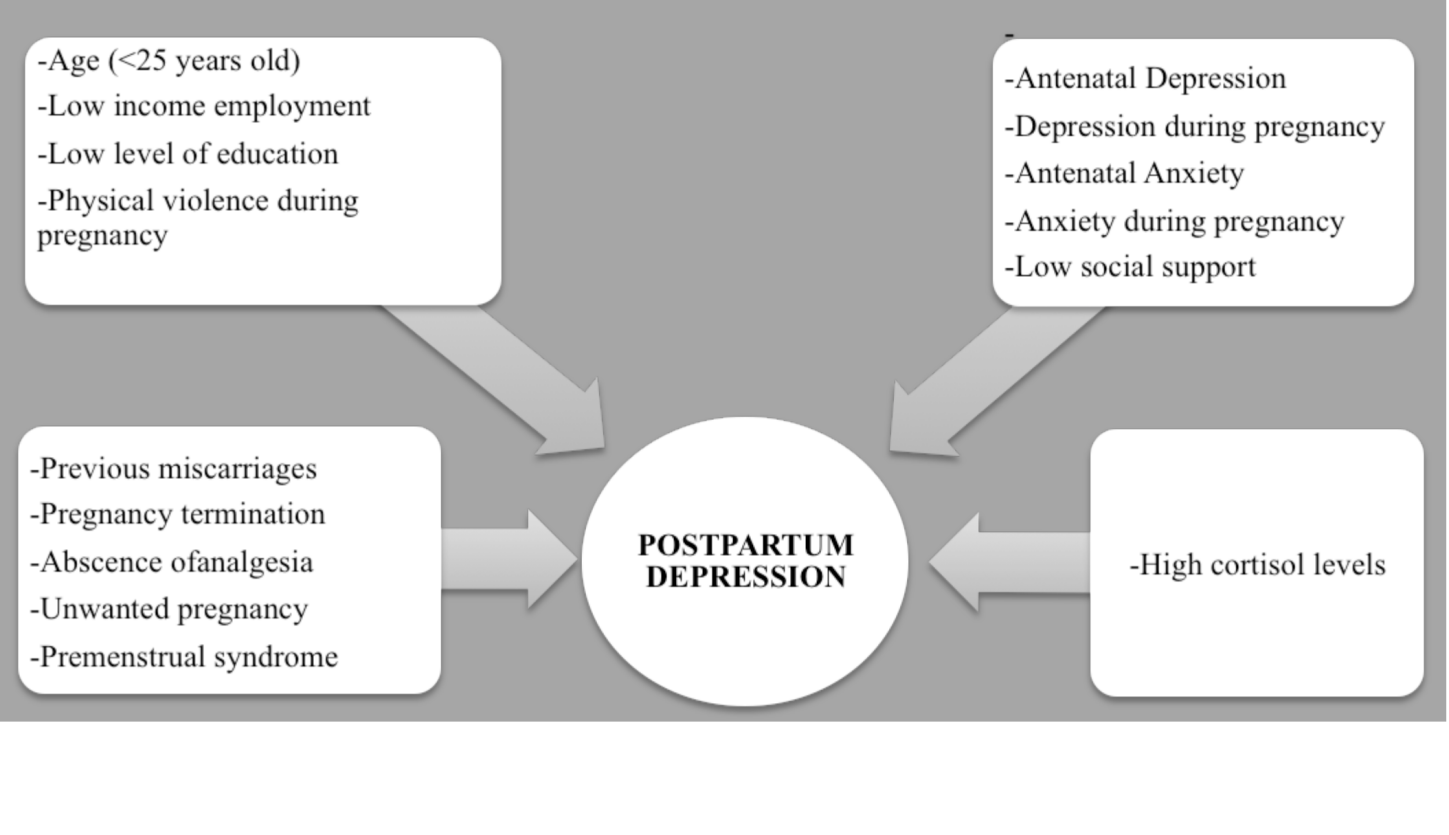
Summary of risk factors during pregnancy associated with postpartum depression [6–13].

It has been previously reported that there is an association between sociodemographic risk factors during pregnancy (e.g. being younger than 25 years old) and postpartum depression symptoms [6, 7]. Furthermore, obstetric risk factors (e.g. previous miscarriages) have been found to be related to postpartum depression [8, 9]. Past histories of psychopathological symptoms or suffering from depression, anxiety, or stress during pregnancy have been reported as psychological variables associated with postpartum depression [10, 11]. Fig 1 shows a summary of risk factors associated with postpartum depression in previous studies.

Another risk factor associated with postpartum depression is the dysregulation of the hypothalamic-pituitary-adrenal axis which results in an increased exposure of pregnant women to cortisol [12, 13]. The hypothalamus synthesizes corticotrophin-releasing hormone (CRH) as part of the biological stress response. CRH stimulates the release of cortisol to prepare the organism to cope with stressful stimuli [14, 15]. Due in part to the presence of the placenta, the hypothalamic-pituitary-adrenal axis is deeply altered during pregnancy. The placenta, a fetal origin endocrine organ, promotes an increased release of cortisol from the adrenal gland through a dramatically increase of placental CRH over pregnancy [12]. Although cortisol negatively regulates the production of CRH from the hypothalamus, cortisol increases the release of placental CRH during pregnancy [12]. Other physical changes during pregnancy include how the pituitary gland doubles its size, and the level of production of cortisol from the adrenal gland increases [14]. Cortisol levels have been generally assessed from urine, saliva, blood, or amniotic fluid samples in pregnant women [16, 17]. Though each matrix offers information about the stress levels the women were experiencing at the time the sample was taken, these methods of assessment require an invasive technique and can be affected by situational variables or circadian rhythms [14, 18].

Alternatively, testing via hair cortisol levels is an innovative technique that offers a retrospectively chronic stress measure of the preceding 3 months, is not invasive, is not affected by the time of the day, and is easy to transport and preserve [15, 19, 20].

The association between postpartum depression and the activation of the hypothalamic-pituitary-adrenal axis during pregnancy remains a challenge. On one hand, an association between postpartum depression and high hair cortisol levels during pregnancy has been reported [21]. On the other hand, urine and blood cortisol levels were not associated with postpartum depression [2, 22]. However, certain associations have been reported between low blood cortisol levels and postpartum depression [23].

A recent review reported future research should improve the accuracy of cortisol measurements over time and use appropriate tools to assess depression [24]. More studies on risk factors associated with postpartum depression may reduce negative pregnancy outcomes [5, 25]. Predicting those variables related to postpartum depression can improve pregnancy and infant health outcomes through tailored interventions during pregnancy [4].

In this respect, the aim of this study was to analyze sociodemographic, obstetric, and psychological variables along with hair cortisol levels during the first, second, and third trimester of pregnancy that could predict postpartum depression symptoms.

## Materials and methods

### Subjects

Participants were recruited at 3 health centers and a general hospital in the South of Spain, while attending a prenatal appointment. Fifty-seven pregnant women voluntarily agreed to participate in this study. Five women had a spontaneous miscarriage during the first trimester. Seven participants were unable to continue in the study during pregnancy due to lack of time. One participant moved to another city before giving birth. Finally, a total sample of 44 pregnant women was longitudinally assessed during the first trimester (*M* = 12.36 weeks of gestation; *SD* = 3.60), the second trimester (*M* = 25.32 weeks of gestation; *SD* = 3.24), and the third trimester (*M* = 34.94 weeks of gestation; *SD* = 3.34). Assessments took place while participants were attending a prenatal appointment with their midwives (health center) and obstetricians (general hospital). Participants were followed up during a postpartum appointment with their respective health care practitioners (*M* = 15.79 days after birth; *SD* = 9.78) and divided into 2 groups: a group of women with postpartum depression symptoms (n = 16), scoring 10 or greater on the Edinburgh Postnatal Depression Scale, and a group of women with no postpartum depression symptoms (n = 28), scoring below 10 on the Edinburgh Postnatal Depression Scale. We used the cut off of 10, as it is the best cut-off score for European Spanish mothers. This cut off was indicative of highly likely to be suffering from postpartum depression [26].

Inclusion criteria was low-risk pregnant women above 18 years old with proficiency in the Spanish language. Participants were excluded if they presented any pathology before or during pregnancy. To minimize the confounding effect of risk variables, pregnancies with Cushing’s disease, asthma, steroid medication, diabetes, and other conditions known to affect cortisol levels, were excluded.

This study was approved by the Human Ethics Research Committee of the University of Granada (reference 881), the Biomedical Ethics Research Committee and the Ethics Research Committee of the Health Centers, and the hospital where this study was implemented. Moreover, this study followed the guidelines of the Helsinki Declaration (AMM, 2008) and the Good Clinical Practice Directive (Directive 2005/28/EC) of the European Union. Participation was voluntary and an informed written consent document was read and signed by every participant.

### Instruments

#### Sociodemographic and obstetrics data

Demographic information was collected my means of the Pregnancy Health Document [27] since it is the official record of the health of every pregnant woman and her newborn.

#### Biological measures

For the purpose of assessing the activation of the hypothalamic-pituitary-adrenal axis, hair cortisol levels were measured through hair samples proximal to the scalp with a length no greater than 3 cm (assuming an average growth rate of 1 cm/month, a 3 cm segment contains cortisol that has been deposited over approximately the last 3 months). Samples consisting of approximately 150 strands of hair were collected from the posterior vertex of the head [28]. The hair samples were wrapped in a piece of aluminum foil to protect them from light and humidity and they were stored in an envelope at room temperature. Afterwards the hair samples were sent for analysis to the Faculty of Pharmacy at the University of Granada. The hair samples were weighed and ground to a fine powder to break up the hair’s protein matrix and increase the surface area for extraction using a ball mill. Cortisol from the interior of the hair shaft was extracted into HPLC-grade methanol by incubation of the sample for 72 hours at room temperature in the dark with constant inversion using a rotator. After incubation, the supernatant was evaporated until completely dry using a vacuum evaporator and the extract was reconstituted in 150 ul of phosphate buffered saline at a pH of 8.0. The reconstituted sample was immediately frozen at -20°C for later analysis [29, 30, 31].

The cortisol in the hair sample was measured using the a salivary ELISA cortisol kit with the reagent provided following the manufacturer’s directions. Using a salivary ELISA cortisol kit is a validated method to assess hair cortisol levels and is highly positive correlated with liquid chromatograph–mass spectrometry (LC–MS/MS) [31]. The sensitivity of the cortisol ELISA kit is 1.0 ng/ml as reported by the manufacturer and the cross reactivity is as follows: Prednisolone 13.6%, Corticosterone 7.6%, Deoxycosticosterone 7.2%, Progesterone 7.2%, Cortisone 6.2%, Deoxycortisol 5.6%, Pednisone 5.6% and Dexamethasone 1.6%. No cross-reaction was detected with DHEAS and Tetrahydrocortisone.

The intra- and inter-assay variations were analyzed on internal quality controls used for routine salivary cortisol measurement, measured in duplicate on eight consecutive assays. The intra-assay coefficients of variance (CV) were 2.7% at 10.7 ng/ml and 4.3% at 43.9 ng/ml. The inter-assay CVs were 4.4% and 6.3%, respectively.

#### Maternal perceived stress

Psychological stress was assessed by means of the 14-item Spanish version of the Perceived Stress Scale (PSS) [32] to evaluate the perception of general stress during the preceding month. Each of the 14 items scores on a 5-point Likert scale (0 = never, 1 = almost never, 2 = once in a while, 3 = often, 4 = very often). The Cronbach’s alpha reliability coefficient of the Spanish version is α = 0.81 [33].

#### Psychopathological symptoms

In this respect, the Spanish version of the SCL-90-R [34, 35] was used to assess psychopathological symptoms. This 90-item scale is scored using a 5-point Likert scale from 0 (never) to 4 (extremely). This instrument is used to assess 9 dimensions: Somatization, Obsession-compulsion, Interpersonal sensitivity, Depression, Anxiety, Hostility, Phobic anxiety, Paranoid ideation, and Psychoticism. The scale also has 7 extra items distributed among 3 global indexes of distress: the GSI, which measures overall psychological distress; the PSDI, which is used to measure the intensity of symptoms; and Positive Symptom Total, used to measure the number of self-reported symptoms. The Cronbach’s alpha reliability coefficients of the Spanish version range are between .67 < α < .94 [35].

#### Pregnancy-specific stress

For this purpose, the Spanish version of the Prenatal Distress Questionnaire (PDQ) [36, 37] was used to assess pregnancy-specific stress. It is a 12-item instrument scored on a 5-point Likert scale from 0 (none at all) to 4 (extremely) to assess specific worries and concerns pregnant women experience regarding medical problems, physical symptoms, body changes, labor, childbirth, relationships, and the baby’s health. The Cronbach’s alpha reliability coefficient of the Spanish version is *α* = .71 [37].

#### Measurement of postpartum depression

The Spanish version of the Edinburgh Postnatal Depression Scale [38, 39] was used to assess the risk of postpartum depression. This 10-item instrument is scored on a 4-point Likert scale ranging from 0 (as always) to 3 (absolutely not). The best cut-off score for the Spanish version was 10/11 as highly likely to be suffering from postpartum depression [26]. A cut off of 10 was also reported to be useful to screen for a posterior psyquiatric assessment in Spanish sample. Using a cut-off of 10 identified 100% of women with major depression, resulting in a combined sensitivity of 79%, specificity of 95%. Although some authors recommend a 12/13 cut-off [38, 40, 41] even in Spanish Americans [42], the sensitivity for depression decreased to 62% and 14% of women with a major depression remained undiagnosed in European Spanish women during the postpartum period [26]. A study lead by the Postpartum Depression: Action Towards Causes and Treatment (PACT) Consortium also used a cut-off of 10 to capture a wider range of severity of postpartum depression (minor to severe) [42]. The Cronbach’s alpha reliability coefficient of the Spanish version is *α* = .79 [43].

### Procedure

Pregnant women attending antenatal appointments at 3 public health centers in Granada and Roquetas de Mar, Spain, and a general hospital in El Ejido, Spain, (September 2015-July 2016) completed a battery of self-report questionnaires during their first, second, and third trimesters of pregnancy, and during the postpartum period. Participants received informative leaflets and stated their intention to participate at the next prenatal appointment. In our context, pregnant women attend an appointment with a General Practitioner (GP) before visiting a midwife. Clinical interviews performed by GPs reflected an absent of any decisional impairment that could affect their capacity to consent [44]. Following the written consent, hair samples were obtained by a specifically trained midwife, according to suitable guidelines and participants completed all 3 previously mentioned questionnaires (PDQ, PSS, SCL-90-R) at home during each trimester, and returned the questionnaires at their next antenatal appointment. The Depression sub-scale of the SCL-90-R was used to assess antenatal depression during pregnancy. Information regarding sociodemographic and obstetric data was obtained at the first antenatal appointment.

After delivery, participants attending a postnatal appointment (*M* = 15.79 days after birth; *SD* = 9.78) with a midwife at a public health center were assessed with the Edinburgh Postnatal Depression Scale.

### Statistical analysis

Firstly, participants were divided into 2 groups: a group of women with postpartum depression symptoms (n = 16), scoring 10 or greater in Edinburgh Postnatal Depression Scale, and a group of women with no postpartum depression symptoms (n = 28), scoring below 10 in Edinburgh Postnatal Depression Scale. In order to verify both groups were equivalent in terms of the main sociodemographic, obstetric, and hair characteristics, 2-sample *t*-tests and a chi-square test were used to compare sociodemographic, obstetric, and hair characteristics between groups.

A mixed 2 × 3 analysis of ANOVA was conducted to check for statistically significant differences between both groups. The first factor includes two levels (women with postpartum depression symptoms and women with no postpartum depression symptoms) between the independent groups. The second was a repeated-measures within-subject factor during three trimesters: 1st trimester (hair cortisol, psychopathological symptoms and stress); 2nd trimester (hair cortisol, psychopathological symptoms and stress); 3rd trimester (hair cortisol, psychopathological symptoms and stress). The Greenhouse-Geisser correction was applied in the repeated-measures analyses. When a significant Group x Sampling Time interactions was found, *Bonferroni* analysis was conducted to determine the trimesters there were differences between. *Bonferroni* analysis is a conservative post hoc procedure designed to compare different combinations while controlling the overall Type I error rate (α) [45]. A follow-up Student’s *t*-test was conducted to determine whether there were differences in cortisol, psychopathological symptoms and stress levels between both groups Due to the fact that hair cortisol levels did not have a normal distribution, a natural log transformation (natural log; *LN* base e) was done.

Previous studies reported subjects with dyed hair presented lower hair cortisol levels [28, 46, 47]. Participants with dyed hair were not excluded. Consequently, we controlled this factor on analysis. A previous study found that fetal sex could affect maternal cognition [48]. For this reason, a 2-sample *t*-test was conducted to test whether the sex of the fetus could influence cortisol levels.

Finally, with the purpose of testing which hair cortisol levels (first, second or third trimester) best explained the Edinburgh Postnatal Depression Scale scores, we carried out a multiple regression analyses using the introduction method. The independent variables were the hair cortisol levels on the first, second and third trimester. The dependent variables were the Edinburgh Postnatal Depression Scale scores.

Data analyses were performed using Statistical Package for Social Sciences 20.0 Mac version (SPSS, Armonk, NY). Differences were considered significant when *p* < .05.

## Results

### Descriptive sample characteristics

A total sample of 44 low-risk pregnant women between the ages of 24 and 39 years old (*M* = 32.38; *SD* = 3.96) participated in this study. As shown in Table 1, *t*-tests and a chi-square test indicate no differences between groups in respect to main sociodemographic data, obstetrics, and hair characteristics. Due to significant differences were found between groups on previous miscarriages (*X*^2^ = 4.71, *p* < .05) and the sex of the fetus (*X*^2^ = 6.03, *p* < .05), we included these variables as covariates on further analysis.

**Table 1:** Differences in sociodemographic, obstetrics variables and depression symptomatology between women with postpartum depression and without postpartum depression.

			No depression X(SD)/%	Depression X(SD)/%	Test a	*p*
Socio-demographic variables						
Age			32.11(4.05)	32.94(3.62)	-.67	.*50*
Nationality	Spanish		24(85.70%)	4(25.0%)	7.86	.37
	Immigrant		4(14.3.60%)	12(75.0%)		
Marital status	Single/divorced/widow		10(35.7%)	8(50%)	.86	.35
	Married/cohabitant		18(64.3%)	8(50%)		
Employment situation	Working		23(82.1%)	11(68.8%)	1.04	.31
	Unemployed		5(17.9%)	5(31.2%)		
Occupation	Health		8(28.6.%)	5(31.2%)	-2.57	.79
	Education		7(25.0%)	2(12.5%)		
	Other		3(46.4%)	9(36.2%)		
Level of education	Secondary school		3(42.85%)	4(57.15%)	3.89	.14
	University		23(69.70%)	10(30.30%)		
Sport	Yes		19(67.9%)	7(43.8%)	2.45	.12
	No		9(32.1%)	9(56.2%)		
Pet	Yes		8(28.6%)	8(50%)	2.02	.15
	No		20(71.4%)	8(50%)		
Hair aspect	Nature		13(46.4%)	6(37.5%)	0.33	.56
	Dyed		15(53.6%)	10(62.5%)		
***Obstetric information***						
Primiparous	Yes		20(71.4%)	8(50%)	2.02	.15
	No		8(28.6%)	8(50%)		
Wanted pregnancy	Yes		24(85.7%)	13(81.2%)	1.52	.69
	No		4(14.3%)	3(18.8%)		
Pregnancy method	Spontaneous		22(78.6%)	13(81.2%)	0.45	.83
	Fertility treatment		6(21.4%)	3(18.8%)	4.71	.03*
Previous miscarriages	Yes		4(14.3%)	7(43.8%)	2.77	.78
Labor and delivery	No		24(85.7%)	9(56.2%)		
	Eutocic		20(74.1%)	9(56.2%)		
	Dystocic		4(14.8%)	2(15.4%)		
	C-section		3(11.1%)	1(7.7%)		
Pain relief in labor	None		2(8.0%)	3(23.1%)	4.11	.13
	Epidural		18(72.0%)	10(76.9%)		
	Warm bath		5(20.0%)	0(0%)		
Sex of the fetus	Female		7(25%)	10(62.5%)	6.03	.01*
	Male		21(75%)	6(37.5%)		
**Depression**						
Antenatal depression	Depression subscale clinical scores (> 70)	1^st^ trimester	4(36,36%)	1 (10%)	0.65	.41
		2^nd^ trimester	2(18,18%)	4(40%)	2.75	.09
		3^rd^ trimester	5(45,45%)	5(50%)	1.04	.30
Postnatal depression	EPDS	< 10 scores	28(100%)	3(18.8%)	32.29	.001*
		10–12 scores	0(0%)	5(31.2%)		
		>12 scores	0(0%)	8(50%)		

Note. Significant at the * = p ≤, 05.

^a^ T-test of students used to quantitative variables and Chi-square test to categorical variables. Sport is presented to inform whether participants practiced or did not practice any regular physical activity during pregnancy.

### Associations of maternal postpartum depression symptoms with indicators during pregnancy

We examined the associations of maternal postpartum depression symptoms with pregnancy-specific stress, perceived stress, and psychopathological symptoms during the first, second, and third trimester. The group with postpartum depression symptoms had higher scorers on the Edinburgh Postnatal Depression Scale (*M* = 13.50; range = 10–24) than the group with no postpartum depression symptoms (*M* = 4.75; range = 2–8), *t* = 9.92, *p* < .001. Five participants scored in the range of 10–12 on the Edinburgh Postnatal Depression Scale (see Table 1).

Regarding the between group analysis, an interaction effect was found between women with postpartum depression symptoms and women with no postpartum depression symptoms on pregnancy-specific stress throughout the three trimesters, *F*(1, 41) = 4.08, *p* = .05, which remained significant when including previous miscarriages, sex of the fetus and antenatal depression as covariates, *F*(1, 37) = 5.67, *p* < .05). *Bonferroni post hoc* analysis on the pregnancy-specific stress revealed no significant differences during the first, second or third trimester on the any of the two groups. Although pregnancy-specific stress levels were higher among participants with postpartum depression symptoms at the first, second, and third trimester of pregnancy, significant differences between both groups were found during the third trimester regarding pregnancy-specific stress (*t* = -2.67, *p* = .01) (see Table 2).

**Table 2:** Mean differences on stress and psychopathological symptoms with interaction effects between groups*trimesters.

Trimester	Questionnaires	Subscalesa	No depression X(SD)	DepressionX(SD)	*t*	*p*
Trimester 1	PDQ		13.96(6.44)	15.81(5.64)	*-1*.*79*	.*08*
	SCL-90-R	SOMS	57.14(26.29)	70.66(19.33)	*-2*.*70*	.01*
		DEP	43.04(23.13)	44.28(18.79)	*-*.*89*	.*37*
		ANX	54.63(28.19)	60.01(17.95)	*-*.*74*	.*46*
		GSI	53.66(25.24)	57.59(19.83)	*-*.*53*	.*59*
Trimester 2	PDQ		12.57(5.54)	14.25(4.41)	*-1*.*03*	.*31*
	SCL-90-R	SOMS	41.90(17.78)	55.96(21.39)	*-2*.*34*	.02*
		DEP	30.04(18.71)	47.33(23.60)	*-2*.*67*	.01*
		ANX	40.41(21.06)	62.72(23.78)	*-3*.*22*	.002*
		GSI	41.22(21.78)	58.22(25.16)	*-2*.*38*	.02*
Trimester 3	PDQ		11.25(4.36)	14.75(3.78)	*-2*.*67*	.01*
	SCL-90-R	SOMS	64.09(29.68)	74.42(27.29)	*-2*.*01*	.*051*
		DEP	44.24(27.15)	59.60(22.79)	*-1*.*90*	.*06*
		ANX	55.50(26.58)	66.43(25.89)	*-1*.*32*	.*19*
		GSI	55.63(29.83)	68.44(27.21)	*-1*.*41*	.*16*

Note.

* Significant at the p ≤ .02 level

^a^PDQ = Prenatal Distress Questionnaire; SCL-90-R = Symptom CheckList 90 Revised; SOMS = Somatisation; DEP = Depression; ANX = Anxiety; GSI = Global Severity Index.

In respect to perceived stress, no significant interaction effect was found between both groups.

**Fig 2:**
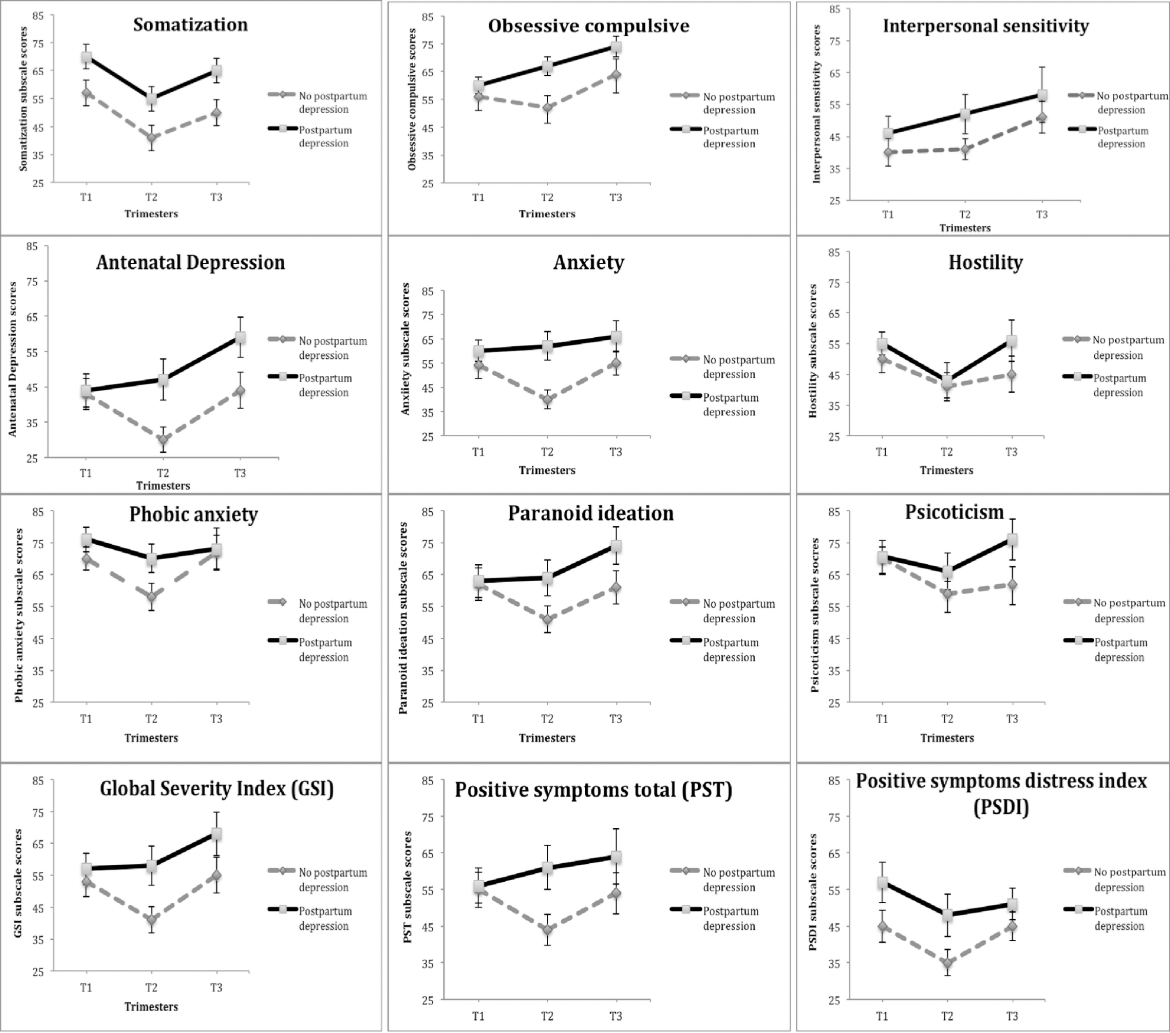
SCL-90-R scores throughout pregnancy in both groups. *Note*. SOMS = Somatization; OBS = Obsessive-compulsive; SEN = Interpersonal sensitivity; DEP = Depression; ANX = Anxiety; HOS = Hostility; PHOB = Phobic anxiety; PAR = Paranoid ideation; PSI = Psychoticism; GSI = Global severity index; PST = Positive symptoms total; PSDI = Positive symptoms distress index; PPD = Postpartum depression; NO PPD = No postpartum depression.

Regarding psychopathological symptoms, the group with postpartum depression symptoms scored higher in every single SCL-90-R subscales during the first, second, and third trimester of pregnancy. This group had clinical scoring (score above 70) in the Somatization, Phobic anxiety, and Psychoticism subscales during the first trimester; Phobic anxiety sub-scale during the second and third trimester; Somatization, Obsessive-compulsive, Paranoid ideation and Psychoticism subscales at the third trimester (see Fig 2).

The number of participants with clinical scores (above 70) on the Depression subscale of the SCL-90-R for both groups in the first, second and third trimester are displayed in Table 1.

Regarding the psychopathological symptoms subscales, an interaction effect between groups on the Somatization subscale, *F*(1, 42) = 6.95, *p* < .05, which remained significant when controlling for previous miscarriages, sex of the fetus and antenatal depression in analysis, *F*(1, 37) = 8.54, *p* < .05. Several repeated measures ANOVA revealed interaction of group*trimester on the Depression subscale, *F*(1, 42) = 3.14, *p* <. 05, which remained partially significant when including previous miscarriages, sex of the fetus and antenatal depression as covariates, *F*(1, 37) = 2.99, *p* = .059; Anxiety subscale, *F*(1, 42) = 4.21, *p* <. 05, even when controlling for previous miscarriages, sex of the fetus and antenatal depression in analysis, *F*(1, 37) = 8.79, *p* <. 05, and the GSI subscale after including previous miscarriages, sex of the fetus and antenatal depression as covariates, *F*(1, 37) = 5.10, *p* < .05. Scales showing significant interaction effect group*trimester mean differences on psychopathological symptoms are displayed in Table 2. Regarding the postpartum depression symptoms group, the pairwise comparisons in for the main effect of trimesters using *Bonferroni* analysis showed significant differences on the Somatization subscale between trimester 1 and 2 (*p* <. 05), and trimester 2 and 3 (*p* <. 05); regarding the Depression subscale significant differences between trimester 2 and 3 (*p* <. 05); in respect to the GSI subscale significant differences between the trimester 2 and 3 (*p* <. 05). No significant differences were found in the no postpartum depression group throughout pregnancy.

Significant differences between both groups were found during the first trimester regarding the Somatization subscale (*t* = -2.70, *p* = .01); during the second trimester regarding the Somatization subscale (*t* = -2.34, *p* = .02), the Depression subscale (*t* = -2.67, *p* = .01), the Anxiety subscale (*t* = -3.22, *p* = .002) and the GSI global index (*t* = -2.38, *p* = .02). As shown in Table 2 psychopathological measures are higher within the group with postpartum depression symptoms. No significant differences were found between both groups during the third trimester.

### Association between hair cortisol levels with postpartum depression symptoms

We examined the associations between hair cortisol levels during the first, second, and third trimester with postpartum depression symptoms. We found the group with postpartum depression symptoms obtained higher hair cortisol levels during the first, second, and third trimesters. A repeated measures ANOVA revealed a significant interaction between trimester*postpartum depression symptoms group on hair cortisol levels, *F*(1, 41) = 7.96; *p* < .001, which remained significant when including previous miscarriages, antenatal depression, sex of the fetus and dyed hair in the model, *F*(1, 36) = 7.78; *p* < .001.

In the group with postpartum depression symptoms, the pairwise comparisons for the main effect of trimesters using *Bonferroni* analysis showed a significant difference between trimesters 1 and 3 (*p* < .01), and a significant difference between trimesters 2 and 3 (*p* < .001). No significant differences were found in this respect in the group with no postpartum depression symptoms.

**Fig 3:**
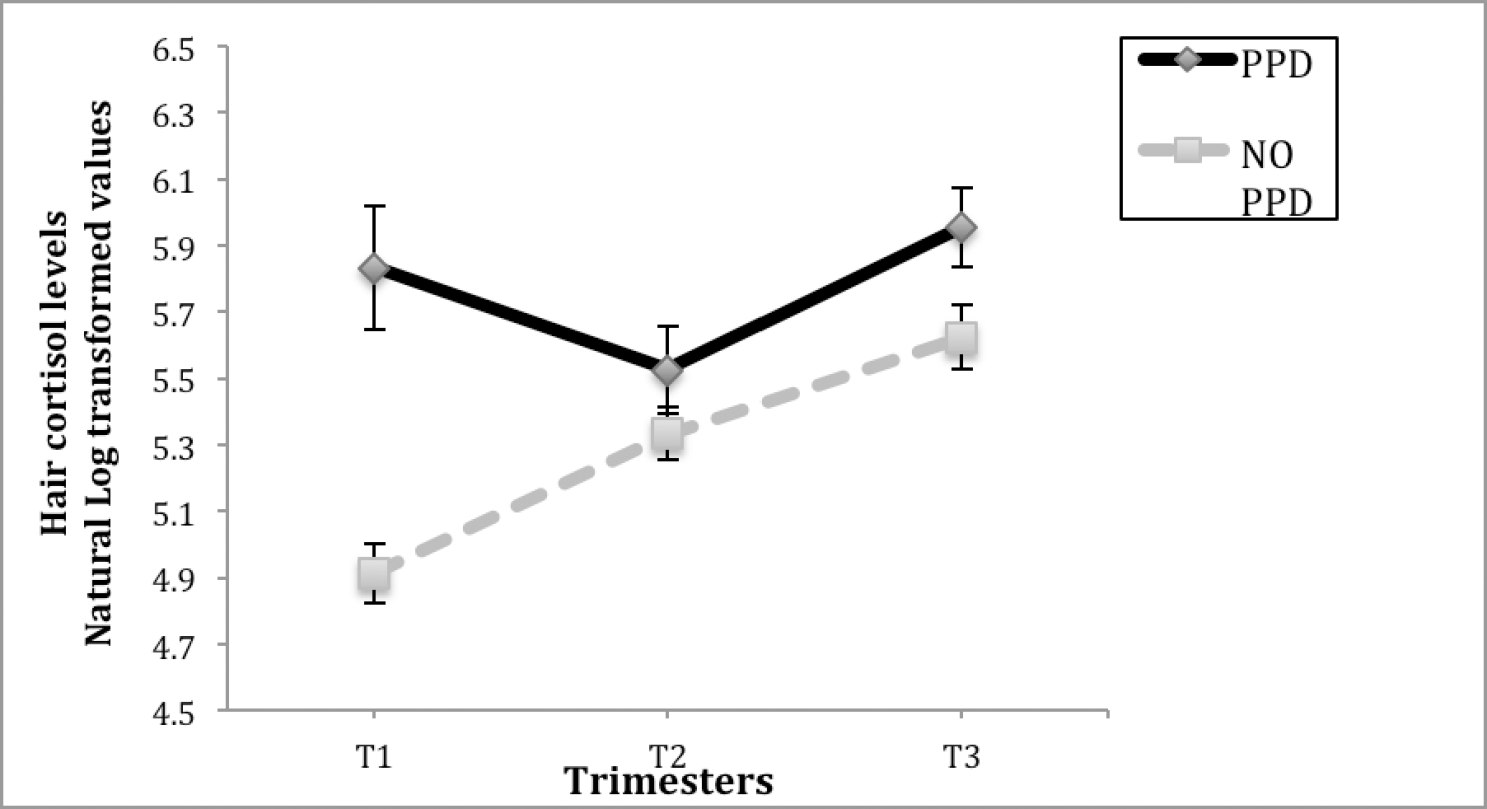
Hair cortisol levels differences (pg/mg) in each trimester between women with and without postpartum depression symptoms. *Note*. *Significant at the *p ≤* .05 level.

A 2 sample *t*-test revealed significant differences between groups regarding hair cortisol levels in the first trimester (*t* = -4.77; *p* < .001) and the third trimester (*t* = -2.06; *p* ≤ .045). No significant differences were found between groups in the second trimester (see Fig 3).

A linear regression was carried out to test whether the mothers’ hair cortisol levels could predict postpartum depression symptoms. Results of the regression revealed that hair cortisol levels could predict 21.7% of the variance of postpartum depression symptoms [R^2^ = .21, (F = 3.703, *p* < .05)]. More precisely, hair cortisol at the first trimester (*ß* = 0.32, *p* < .05) and the third trimester (*ß* = 0.32, *p* < .05) significantly predicted the Edinburgh Postpartum Depression Scale scores.

A 2-sample *t*-tests was used to assess whether the sex of the fetus could influence the release of cortisol during pregnancy. The independent variable was the sex of the fetus and the dependent variable were hair cortisol levels during the first, second and third trimester. No significant differences were found (*p* > .05) (see Table 3).

**Table 3:** Maternal hair cortisol levels and sex of the fetus.

Trimesters	Female fetus *X*(*SD*)	Male fetus *X*(*SD*)	*t*	*p*
1st trimester	5.49(.94)	5.08(.56)	-1.81	.07
2nd trimester	5.30(.55)	5.46(.40)	1.11	.27
3rd trimester	5.67(.54)	5.38(.52)	.69	.49

Note. Hair cortisol levels are log transformed values

As shown in Fig 3, hair cortisol levels increased from the first to the third trimester in the group with no postpartum depression symptoms, getting the higher hair cortisol levels at the third trimester. Nonetheless, in the group with postpartum depression symptoms, hair cortisol levels decreased from the first to the second trimester, and increased in the third trimester.

## Discussion

The aim of this study was to study the sociodemographic, obstetric, psychological, and hormonal variables that may predict postpartum depression symptoms. For this purpose, we compared a group of pregnant women with postpartum depression symptoms with a group of women with no postpartum depression. Sociodemographic variables have been informed to be relevant when assessing the psychological wellbeing of women in the postpartum period [49–51]. Significant differences were found between groups in respect to previous miscarriages and the sex of the fetus. Therefore, these variables were included as covariates in further analysis. The group with postpartum depression symptoms had higher pregnancy specific stress, perceived stress, psychopathological symptoms and hair cortisol levels during the three trimesters of pregnancy.

However, significant differences were found in this study in respect to psychopathological symptoms during the first and second trimester, and in respect to pregnancy-specific stress during the third trimester between both groups. More precisely, during the first trimester, significant differences were found in respect to the Somatization SCL-90-R subscale. During the second trimester, significant differences were found in respect to Somatization, Obsessive-compulsive, Depression, Anxiety, IGS, and Positive Symptom Total subscales. Accordingly, it has been reported high correlations between the Anxiety and Somatization SCL-90-R subscales during the first and second trimester and the Edinburgh Postnatal Depression Scale [52]. In this respect, recent studies reported the presence of psychopathological symptoms through the SCL-90-R during pregnancy to be related with postpartum depression [53, 54]. More specifically, depression, anxiety, and stress during pregnancy have been related with negative pregnancy outcomes, including postpartum depression [55, 56]. Psychological stress during pregnancy has been related to postpartum depression [57]. We could not find any significant differences between groups regarding perceived stress at any time point. These findings do not agree with those reported by Scheyer and Urizar [58] who reported significant association between perceived stress during the 3 trimesters of gestation and postpartum depression. We hypothesize this could be due to the PSS being a general stress measure and that pregnancy-specific measures may likely improve pregnant women’s psychological assessments. In this regard, pregnancy-specific stress was significantly different between groups during the second trimester. The fact that although both stress measures (PDQ and PSS) have been widely used when assessing stress levels during pregnancy, is noteworthy that the PDQ offers the opportunity to assess specific worries and concerns related to pregnancy and therefore is a more consistent pregnancy-specific stress measure and a better predictor of negative pregnancy outcomes [55, 59].

Biochemical measures as those provided through hair cortisol levels inform of chronic stress levels [29]. According to our results, hair cortisol levels in the group with postpartum depression symptoms descended from the first to the second trimester and ascended from the the second to the third trimester, resembling an U shape when plotted on a graph. This is the first study to report hair cortisol levels throughout pregancy in a group of women with postpartum depression sypmtoms. Although an upward course regarding cortisol levels throughout pregnancy has been previously reported [60], these findings were reported in respect to pregnant women with no postpartum depression symptoms.

In the present study, hair cortisol levels were higher in the group with postpartum depression symptoms compared to the group with no postpartum depression symptoms in the 3 trimesters and were significantly different in the first and the third trimester. A previous study reported that cortisol levels during the second trimester, but not in the third trimester, were higher and significantly different in those women with postpartum depression [61]. Our findings in this regard support the fact that high stress levels during pregnancy are related to postpartum depressive symptoms [62, 63]. In fact, hair cortisol levels predicted postpartum depression symptoms in our study. More precisely, hair cortisol at the first trimester and the third trimester could predict postpartum depression symptoms. Our results do not support other studies reporting low cortisol levels during pregnancy with postpartum depression [24, 58]. We hypothesize these differences may be due to the fact that previous studies have used acute stress biological measures (e.g. urine cortisol levels), and in our study hair cortisol samples were used which reflects levels of chronic stress within the last 3 months [15, 19, 20]. Our study has several strenghts. First, the longitudinal design of our study offered an unique possibility of observing the range of psychological symptoms, including prenatal stress and cortisol levels throughout pregnancy in both groups. Second, we have used the PDQ, a pregnancy-specific stress measure which shows a consistent relation with negative pregnancy outcomes [55]. More importanltly, the innovative assessment of stress through hair cortisol levels gives evidence of chronic stress through a single measurement [19]. Several studies have shown the benefits of knowing the risk factors involved in postpartum depression to improve maternal and infant outcomes [2, 4]. Finnally, we considered the influence of a variety of sociodemographic and obstetric variables that have been previously associated with pospartum depression [6–9]. It is important to note that this study focused on assessing the association between sociodemographic and obstetric variables, psychological stress, psychopathological symptoms, and biological stress measured through hair cortisol levels, with postpartum depression symptoms. The percentage of women in the postpartum depression group (36%) in our study is quite high with respect to previous studies reporting percentages of 10–15% [2]. In our context, it exits a lack of clinical screening and prevention related to postpartum depression, which might lead to greater numbers of women with postpartum depression symptoms. Nevertheless, the prevalence of postpartum depression can vary between studies, due to the different criteria used to define postpartum depression [13].

Although participants were longitudinally and prospectively assessed throughout pregnancy and the postpartum period, a limitation of the present study is the relatively small sample size, which should be considered for the interpretation of the data. A further potential limitation was that postpartum depression symptoms were only assessed at a single time point. A second follow-up after delivery would have offered the possibility to evaluate the participants’ long-term psychological wellbeing and study possible associations with health variables during pregnancy.

In summary, according to our findings, high levels of maternal stress during pregnancy are associated with postpartum depression symptoms. Psychopathological symptoms in the first and second trimester, high pregnancy-specific stress in the second trimester, and high hair cortisol levels in the first and the third trimester were associated with postpartum depression symptoms. Since hair cortisol levels reflect stress levels during the previous 3 months preceding the time the sample was taken [20], our findings reflect that the preconception period, and the second trimester of pregnancy, are particularly sensitive periods related to postpartum depression symptoms. Our findings do not agree with a previous study reflecting changes in the HPA related with postpartum depression occur during the postpartum period [64] instead, we found changes in the HPA may begin during the preconception period. In line with our findings, a recent study reported higher cortisol levels during pregnancy were related to postpartum depression symptoms [13]. In this respect, prenatal effective stress screening interventions should be used widely during this time to reduce adverse outcomes [65]. These findings are of clinical and research importance since they show the health variables that can predict postpartum depression symptoms among pregnant women. Assessing psychological health in the perinatal period can aid practitioners in making adequate decisions and provide valuable data on maternity care [66].
